# Pharmacologia; Being an Extended Inquiry into the Operations of Medicinal Bodies, upon Which Are Founded the Theory and Art of Prescribing

**Published:** 1843-07

**Authors:** 


### Art. XII.

*Pharmacologia; being an extended Inquiry into the Operations of Medicinal Bodies, upon which are founded the Theory and Art of Prescribing.* By J. A. Paris, m.d. Cantab. f.r.s. &c. *Ninth Edition, rewritten, in order to incorporate the latest discoveries in Physiology, Chemistry, and Materia Medica.—London,* 1843. 8vo, pp. 622.

The author commences his work with an elegant oration on the Revolutionary History of the Materia Medica; and gives a graphic description of the twin sisters Superstition and Credulity.

> "Credulity," he says, "has been justly defined belief without reason. Scepticism is its opposite, reason without belief, and is the natural and invariable consequence of credulity; for it may be generally observed that men who believe without reason are succeeded by others whom no reasoning can convince; a fact which has occasioned many extraordinary and violent revolutions in the materia medica, and will explain the otherwise unaccountable rise and fall of many useless as well as important articles.........By bestowing unworthy and exaggerated praise upon a remedy, we in reality do but damage its reputation, and run the risk of banishing it from practice; for, when the sober practitioner discovers by experience that a medicine falls so far short of the efficacy ascribed to it, he abandons its use in disgust, and is even unwilling to concede to it that degree of merit, to which in truth and justice it may be entitled; the inflated eulogiums bestowed upon the operation of digitalis in pulmonary diseases excited for a time a very unfair impression against its use. It is also well known with what earnestness the profession regarded the expectations raised by Störk, of Vienna, in 1760, with regard to the efficacy of hemlock.........In the earlier editions of this work, I predicted the fate of the cubebs, which had been restored to notice with such extravagant praise and unqualified approbation; who now places any confidence in the specific powers of that substance in the cure of the disease for which it was considered a never-failing remedy? May the advocates for the virtues of iodine, and of other more recently introduced remedies, derive a useful lesson of practical caution from these precepts!" (pp. 38-9.)

We give Dr. Paris full credit for his prophetic spirit, and agree with him that the remedial powers of both of the last-mentioned substances have been grossly exaggerated. We also entirely coincide with him in the observation which follows, and the truth of which we shall plead in justification of the manner in which we shall treat his own work :

> "As we are investigating the follies of physic, it will not be foreign to the subject, to observe that the above remarks may, with as much truth and force, be applied to medical writings as to medical substances; nothing is more fatal to the permanent character of an author than the extravagant and unmerited encomiums of the reviewer, superlatively lavished on inferior claims." (p 39.)

Under the head "false theories and absurd conceits" the homœopathists are ably dealt with, and treated according to their deserts. The subjects of the "application and the misapplication of chemical science" "devotion to authority and established rule, &c." are next ably discussed, and are followed by a discussion on the "ambiguity of nomenclature." In this chapter the author has made some rather caustic remarks upon the Edinburgh Pharmacopœia. We are fortunately not an interested party in this controversy, and can therefore regard the dispute without feelings of prejudice or prepossession. In a former Number we stated the grounds upon which we preferred a national pharmacopœia in the vernacular tongue, to that of one written in a dead language, and we have met with nothing in the arguments of Dr. Paris to shake our belief in the preponderating advantages of the latter. And although we by no means approve of several of the names adopted in the Edinburgh Pharmacopœia, it is almost a self-evident proposition that permanency of name will secure the greatest accuracy in the preparation of prescriptions. In corroboration of this view we may quote the author's own admission :

> "In our state of transition from the former to the present pharmacopœia, the practitioner has occasionally fallen into the error of employing the nomenclature of both in the same prescription, indiscriminately mixing up that of 1824 with that of 1836. If he thinks proper to adhere to the term hydrargyri submurias, all the names associated with it ought to be contemporary ; in which case his language, to whichever standard it might refer, would at least, be consistent and intelligible. I have been informed by dispensing chemists that a departure from this rule has been attended with considerable embarrassment, and might lead to serious mistakes." (pp. 467-8.)

It however appears to us that, independently of the merits of the question, the author would have shown more wisdom in withholding controversial matter of this kind, from a work destined for the instruction of students and junior practitioners.

The "progress of physiological botany" is shortly noticed, and the chapter on the "influence of soil, culture, climate, and season," is comprehensive and full of valuable instruction. The introductory part also contains a few sensible remarks upon the "unseasonable collection of vegetable remedies, &c."

The Second Part of the work is devoted to the consideration of "*The Operation of Medicinal Substances, and the Classifications founded on them.*" Much has been written upon the classification of the materia medica, and many arrangements have been proposed, but hitherto no plan free from objections has been brought forward. For practical purposes, a physiological or therapeutical arrangement, or a judicious combination of these two, possesses the greatest advantages, as it places before the practitioner, in one view, all the available remedies employed in the cure of certain diseases, or to excite the action of certain functions. Dr. Paris mentions, with approbation, the classification of Dr. Cullen, but adopts that of the late Dr. Murray, whose admirable work on Materia Medica continued for so many years unrivalled by any contemporary publication on this subject. With additions, the author considers it well calculated to furnish a "frame-work" for the display of those therapeutical distinctions, the knowledge of which is essential for the successful administration of remedies and the full comprehension of his practical doctrines.

STIMULANTS. According to Dr. Paris, the term stimulant is very vague and indefinite, and hence is productive of very injurious opinions and practices, but as his views are peculiar, we shall give his own words :

> "In its popular and generally accepted meaning, it [a stimulant] denotes any influence which accelerates the vital movements of the sanguineous and nervous systems, and is thereby supposed to exalt the energies of the body ; but action is not power. In the first place let it be observed that, with the exception of what have been justly termed 'vital stimuli,' such as food, air, water, sleep, and heat, the whole range of the materia medica does not furnish a single agent which is capable of directly increasing the energies of the body, or of adding to the general stock of vital power.......... Our misconception of the term has arisen from a partial and superficial view of the immediate effects of a few limited agents, of which brandy may be taken as their type; it produces a temporary excitement of the arterial and nervous systems, but this is invariably followed by a corresponding depression: all that we have done then is to disturb the balance; we have added nothing to the general amount of power."(pp.169-70.)

It may be true that the materia medica furnishes few agents capable of directly and permanently increasing the vital powers; but the researches of modern physiologists and the general experience of mankind, fully prove that alcohol and alcoholic liquors are capable of assimilation, or are alimentary bodies, containing a considerable amount of carbon, and applicable either to the support of certain tissues, or for the supply of this element to the lungs and skin, during the processes of respiration and perspiration. How else can be explained the tendency to obesity which is induced in the individuals who indulge in their use? The following passages will further illustrate the author's views:

"There is scarcely a remedy, therefore, that might not properly fall under the designation of a stimulant; thus, congestion may take place in a nervous centre, and the whole system, in consequence of the oppression, exhibit symptoms of declining power; in such a case, venesection is a stimulant. The two most essential processes of animal life are nutrition and excretion, and these are exclusively performed by capillary vessels; suppose the balance of their circulation to be disturbed; mercury, by restoring and equalizing it, might, in certain cases, prove a true stimulant. The body may also exhibit symptoms of languor from intestinal and biliary accumulation; under such circumstances, a purgative assumes the character of a stimulant. The nervous system may be in a condition that repels sleep; a judicious and well-directed narcotic, inasmuch as it affords the means of indirectly giving power to the body, may be correctly considered as a stimulant." (p. 170.)

Although there is much truth in the foregoing remarks, the principles inculcated are by no means very definite, and they afford as much if not more scope for the construction of false and dangerous theories in practice as the ordinary definition he complains of, which, in our belief, is nearer the truth than his own. Dr. Paris considers it a great defect in the classification of Dr. Murray, that no distinct place is assigned to exhilarants, which is one of his additions. With this opinion we do not agree; for those substances possessed of the properties he enumerates are aromatics, the active constituents of which are essential oils, and they differ little in their action from the ordinary vegetable tonics, and when they operate as exhilarants, which is not frequently the case, it is generally by exciting an agreeable impression on the digestive organs.

NARCOTICS. This order of remedies is exceedingly diversified in action, and its members possess so different physiological and therapeutical properties, that no general principles can very safely be deduced from them. Authors in general, however, when describing their effects upon the various organs of the body, make some allusion to opium or its preparations, thereby showing that the simple narcotics are only referred to. Indeed, we consider it nearly impossible to give anything but a very general description of their effects, such as is given by Dr. Murray. Dr. Paris seems to entertain opinions not more definite:

"In conclusion," he says, "it may be observed that there is probably no class of medicinal bodies, the individuals of which are less disposed to bend and conform to an artificial arrangement; each would seem to have its own particular mode of operation and to affect sensibility in its own peculiar manner, and hence the practitioner will often find that, after the failure of one narcotic, the administration of another will induce sleep or tranquillity." (p. 176.)

For practical purposes, it is very desirable that some author would arrange narcotics into subdivisions, according to their peculiar physiological or therapeutical actions in ordinary doses, and not from their poisonous effects.

Sedatives. There exists much room for dispute on the subject of this class of remedies, as there are no very precise facts upon which a proper theory may be founded. Some authors, however, assert very positively that some substances act primarily as sedatives; that is, never cause excitement of any kind, either in the first or last stages of their operation. An abstract opinion of this kind is of minor importance, but when a certain line of practice is founded upon it, an erroneous treatment may be the result.

"A sedative," according to Dr. Paris, "in whatever dose it may be given, is never followed by the slightest indication of excitement; it directly and primarily depresses the powers of life, whereas a narcotic in small doses never fails to increase the vital force. It therefore appears to me to be practically essential that we should recognize a class of agents so evidently distinct from all others, not for the sake of giving support to a particular theory, but to warn the practitioner against an error of practice, that of combining in the same prescription remedies obviously incompatible, from a belief in the similarity of their operation. Suppose, for instance, our object was to allay irritability by hydrocyanic acid, would it not be inconsistent to combine it with a small dose of opium, or a more considerable one of alcohol or of ammonia?" (pp. 176-7.)

If it were true that the therapeutical virtues of hydrocyanic acid depended on its depressing effects, the reasoning and practice of the author would be correct and conclusive; but as its mode of action in relieving irritability is totally unknown, and, on the most favorable view, purely hypothetical, his deductions cannot be admitted. It must not be imagined, however, that we advocate a combination of hydrocyanic acid with opium, alcohol, or ammonia: on the contrary, we generally exhibit it without the addition of any other medicine, but we have in one or two cases given it in combination with laudanum in cases of vomiting, and we see no reason why a little brandy might not be exhibited occasionally as an adjuvant. Its combination with ammonia does not neutralize its poisonous effects, according to several experimenters, and as stated by Dr. Christison; and why should it counteract its medicinal operation? Is laurel-water less efficacious than hydrocyanic acid because it contains an essential oil? Although, therefore, it may be unsafe to establish a class of medicinal agents upon hypothetical grounds, it may be useful to retain it on account of its practical utility; for though a slight excitement may accompany the primary action of a remedy, its secondary or depressing operation may be equally powerful, as if no stimulation was produced.

Antispasmodics. According to Dr. Paris, antispasmodics are substances supposed to possess the power of allaying the inordinate action of muscular structures. The causes of spasm he considers to be the following: 1, Irritation of the nervous centres; 2, A loss of balance between the nervous and sanguineous system; 3, Irritation in the primæ viæ; 4, Cold; 5, Excessive muscular reaction, excited by over-extension; 6, A laborious effort to expel foreign matter. With the exception of the second, the causes assigned seem to be judicious. It is difficult to comprehend what is meant by a loss of balance between the nervous and sanguineous systems. It is no doubt true that a state of health necessarily implies the mutual cooperation of these two systems, but we have strong objections to such indefinite principles, particularly as no practical rules for ascertaining *the balance* are given, and the student is thus left in the wide field of hypothesis. After stating that narcotics are most important resources in allaying irritation and pain, the author enumerates certain agents which appear to exert a specific control over spasmodic action, independently of any influence upon its exciting causes, such as assafætida, musk, castor, ammonia, camphor, valerian, &c. He does not, however, establish this proposition very clearly, and some of the substances he enumerates have a powerful influence upon the exciting causes; such as ammonia in neutralizing acidity, and in promoting the heat of the body; indeed, all of them have a tendency to excite the calorific functions of the system, by quickening the circulation of the blood, &c. Why is camphor placed among this list, as almost all writers on materia medica consider it a narcotic?

Tonics. The chapter on this class of substances is sound and practical, and some excellent remarks are made upon the injudicious use of tonics in certain affections.

Astringents. This article also contains much sound and useful information; but we pass on to the local or special stimulants.

Emetics. These agents are well defined, and the various views of physiologists are ably discussed. The author here takes credit to himself, and justly, in our opinion, for anticipating the discovery of Magendie, that vascular congestion retards or suspends the operation of the absorbents; Dr. Paris having previously referred to the influence of venesection in accelerating the absorption of mercury.

Purgatives. These substances have three different modes of operation:

1. By exciting the peristaltic motion of the intestines.
2. By stimulating the excretory and exhalant vessels of the inner coat of the intestines.
3. By stimulating the neighbouring viscera, as the liver and pancreas.

The author makes a separate classification of Purgatives and Laxatives; the former being special stimulants, and the latter mechanical remedies. He brings forward bran as an example of the latter operation, and the coarse fibrous grasses eaten by the lower animals. A decoction of bran is, however, known to be laxative in its effects, and it does not appear to us that he has adduced sufficient evidence in support of his opinions that any of the purgatives act purely on mechanical principles.

He agrees in opinion with the greater number of writers on the subject, that different purgatives act upon the different parts of the intestinal canal; as aloes upon the colon and rectum, colocynth upon the whole tube, mercury upon the functions of the liver, saline purgatives in increasing the watery secretions from the mucous surfaces; and, with some show of reason, entertains a liking for the old terms, hydragogues, cholagogues, &c.

"If there be a circumstance in the treatment of disease, which, above every other, is the government of a blind routine, it is the government of the bowels; let the complaint be what it may, the temperament, strength, or circumstances of the patient be ever so different, the first question of the practitioner relates to the bowels, and should they not have acted during the previous twenty-four hours, away he flies to the aid of aloes, colocynth, senna, calomel, &c. &c., to force the reluctant canal to pour forth its contents." (p. 210.)

We entirely agree with the author as to the morbid affection of the profession in this country for purgatives, but see no reason to question the judgment of the practitioner who in every case inquires carefully into the state of the bowels. The doctrines of Dr. Hamilton have often led young practitioners astray in the treatment of some diseases, particularly in typhus and scarlatina. Purgatives are highly beneficial during the early stages of these diseases; but after symptoms of debility begin to manifest themselves, as not unfrequently happens in weak or cachectic individuals, they ought to be used with extreme caution. A regular evacuation of the bowels, is, however, necessary, which may be effected by moderate doses of mild purgatives, or by enemata. An opposite practice, such as is sometimes adopted in the treatment of dothonenteritis is also very injurious to the patient, as the morbid secretions and alimentary matters become a source of irritation, either by sympathy or absorption. The same rule applies more or less to every other disease.

Emmenagogues. The article on remedies of this class is short, but clear and comprehensive. The author has no confidence in the specific action of certain substances on the uterus. Almost all the old specific emmenagogues have been justly consigned to oblivion, but ergot of rye has lately been extolled by some respectable authorities as redeeming the credit of the class. Its action on the uterus during labour is established beyond all dispute, and this action is capable of no other explanation, but that usually applied to diuretics, expectorants, &c., namely, that it exerts a stimulating influence upon the uterus independent of any particular general effect. No other substance is unequivocally known to possess this operation, and hence it is by no means improbable that even in the unimpregnated state, it may have some influence on the uterus, particularly during menorrhagia, when coagulated blood is liable to be formed in its cavity. In our experience, however, it is nearly as uncertain in its operation upon the menstrual secretion as other emmenagogues; but as it is productive of no injury to the constitution, it may, in many cases, be associated with the general treatment.

Diuretics. These remedies are classified by Dr. Paris as follows:

"1. Medicines which act primarily on the urinary organs.

"2. Medicines which act primarily on the absorbents, and secondarily on the kidneys.

"3. Medicines which act primarily on the stomach and primæ viæ, and secondarily on the absorbents." (pp. 214-5.)

The following substances are stated not to undergo any decomposition *in transitu*, namely, potassa, potassæ nitras, oleum terebinthinæ, juniperus communis, cantharides, and potassæ hydriodas; but squills, colchicum, copaiba, broom, &c. are decomposed. Are the experiments conclusive upon which this division is founded particularly regarding the vegetable substances? We strongly suspect that the question is still not correctly determined.

The medicines which act primarily on the absorbents are limited to mercury and iodine; and although it is rather an obscure subject, we are inclined to agree with the author in this view. The first of these agents undoubtedly has a powerful effect in promoting the absorption of several tissues, natural as well as morbid, and during a slight mercurial action, a diminution of weight is observable. The same occurs with iodine when exhibited in large doses, and some authors have accused this medicine of causing the absorption of the mammæ.

Medicines which act primarily on the stomach produce their effects—

1. By diminished arterial action, as in the case of tobacco, digitalis, &c.

2. By increasing the tone of the system. An instructive case is given in illustration, and its application in the acute diseases of old and cachectic individuals ought to be carefully weighed by the practitioner before he abstracts much blood from the system.

3. By producing catharsis. The following is the author's graphic description of the action of this class of remedies:

> "In the whole circle of medicinal operations, there is nothing more wonderful than this, that an impression made on the internal surface of the primæ viæ, by a few particles of matter, should thus convey by magic, as it were, an impulse to the most remote extremities, rousing their absorbents to action; and in cases of œdema, thus awaking the sleeping energies of the vessels, which, like millions of pumps at work, transmit the morbid fluid to the intestines and urinary passages, effecting a detumescence of the hydropic limbs in the course of a few hours, and thus affording a striking illustration of the sympathetic action of medicine, and are instructive examples of the operation of those of the sorbefacient class." (pp. 222-3.)

Diaphoretics. Dr. Paris justly condemns the use of stimulating diaphoretics during febrile and inflammatory diseases. Some authors are of opinion that several fevers, such as typhus, may be checked *in limine* by diaphoretics, like ordinary inflammatory diseases. This opinion appears to be totally without foundation; no physician experienced in the treatment of the latter disease has been able to verify this statement. This misconception arises from confounding other fevers or febrile disorders with typhus, which they intimately resemble in their early features.

Expectorants. The theory of the action of bodies of this class is somewhat obscure. It is highly probable, however, that some expectorants enter the circulation, and in this manner stimulate the bronchial tubes. Dr. Paris enumerates garlic, squills, the different balsams and fetid gums as belonging to this order. Some excellent remarks, worthy of serious application by the practitioner, are made upon atmospheric changes in relation to moisture and dryness.

Passing over Sialogogues and Counter-Irritants, which are treated with the author's wonted cleverness, we come to

Chemical remedies. An elaborate and well written account is given of the modern discoveries and opinions regarding digestion, the properties of the saliva, and the qualities of the blood, &c. Dr. Paris seems to hold the same opinions as Dr. Prout, that the generation of lactic acid is the source of many diseases, by imperfect assimilation. On this hypothesis he explains the advantages resulting from the protoxide of iron, as it has a great affinity for this acid ; and may thus possibly act by clearing out such remains of this impurity as the emunctories have failed to eject. He lauds the mist. ferri comp. in that form of dyspepsia characterized by " a red tongue and pultaceous evacuations resembling pea-soup." (p. 273.)

Refrigerants. It must be confessed with the author that our notions respecting the action of this class of remedies are by no means well established. Many substances supposed to be refrigerant, from some inherent property of their own, undoubtedly owe this quality to the cold water in which they are exhibited. Acids, also, may owe a portion of their cooling properties to their peculiar effects on the gustatory organs, which effects are propagated by sympathy, to the whole system. Thus a small quantity of a very acid juice excites a shiver or slight tremor over the body, similar to the effects produced by sprinkling the forehead suddenly with very cold water. Dr. Paris demolishes the theory of Dr. Murray respecting the refrigerant operation of vegetable acids, fruit, &c., as is often his wont, in one elegant sentence. " Such," he says, "is the philosophical web which chemical ingenuity has wove for us; the device is beautiful, but the fabric will be found too frail to endure the touch." (p. 277.) The theory of Dr. Crawford respecting animal heat is as summarily confuted : " If the heat of the body depended upon respiration alone, any one might, by a voluntary effort of quick, deep and prolonged respiration, increase it at will," &c., (p. 277 ;) but, we suspect, some of our chemical friends could find grounds for still adhering to the theory of Crawford, even although our accomplished author had himself condescended to exhibit before them the inefficacy of the most potent respiratory efforts to warm up his corporeal system.

The theory of Liebig is next examined and his logical reasoning is opposed simply by the following assertion :

> " But the theory of Liebig assigns the chemical change which converts the organic salts into a carbonate, to the absorption of oxygen in the lungs, whereas it is my belief that the vegetable acid, (e. g. the acetic,) is decomposed in the stomach by the ordinary powers of digestion, and that the alkaline base is there eliminated in the state of carbonate, or at least that it acquires carbonic acid during its transit." (p. 280.)

Antacids. The section on this class presents nothing particularly worthy of notice.

Antidotes. The article on this class is full and instructive, and though we are not prepared to approve of the author's classification of poisons, his remarks regarding the treatment, by emetics, decomposition, and consecutive measures, are exceedingly judicious. Dr. Paris seems still to entertain the opinion that the bichloride of mercury or corrosive sublimate is converted into the chloride or calomel by the agency of albumen ; whereas it is now generally stated by chemical authorities that it merely forms an insoluble and inert compound with it, which we believe has been named by Lassaigne the chloro-hydrargyrate of albumen.

"Antiseptics. The term putrefaction with reference to the question before us has been by far too loosely and indiscriminately applied. According to popular acceptation, we understand by it a succession of chemical changes, characterized by a more or less nauseous and offensive smell, by which an organic body, after passing through various stages of softening and attenuation, is ultimately exhaled, leaving only a small and fixed residue of earthy and saline matter. But let it be remembered that, between the incipient motions and those ulterior phenomena to which we have alluded, a number of intermediate changes occur, each of which must at once become final the instant the chemical and actual affinities are brought to a balance. It is therefore to the early links in the chain of decomposition that the attention of the pathologist is to be directed. . . . In those diseases, to which the epithet of *putrid* was formerly given, such as certain fevers, sea-scurvy, &c., the putrescent odour of the discharge, as well as the very rapid manner in which the body, after death, runs through the extreme stages of putrefaction, renders it probable that some of the preliminary decompositions may have commenced previous to the extinction of life. In all our discussions upon this question, it seems desirable that we should adopt some term to express the ascendency of chemical forces consequent upon the decadence or failure of vital power; the term *hyper-chemisis* might denote such a condition, reserving that of putrefaction to express the ulterior stages and consummation of the process." (pp. 308-9.)

The theory of Liebig respecting the operation of contagious matter in generating diseases is next alluded to with approbation, namely, that "a body, the atoms of which are in the act of transformation, (to which he has given the name of the exciter,) may impart its peculiar condition to compounds with which it may happen to communicate." (p. 310.)

The whole phenomena of the contagious fevers are agreeable to this beautiful law; the blood is universally altered; all the secretions are depraved; many of the organs are in a morbid state; and the great similarity in the symptoms, progress, and termination of the cases all tend to prove that the important series of changes which has been produced are the result of a small quantity of transforming matter infused into the system. The author next discusses the means to be employed in preventing the putrefactive process, namely, "by removing the influences necessary to its commencement. . . . . These may be said to be the presence of a certain portion of water, the access of air, a moderate degree of heat, and the contact of some matter, however small in quantity, in which putrefaction has already commenced, or, in the language of Liebig, the atoms of which are in the act of transformation." (p. 313.) Of all the disinfectants, pure air is considered the most efficient; some influence is, however, ascribed to chlorine.

Antilithics. The article on this subject is full of modern information, and is worthy of careful perusal; but as many of the remarks are taken from Dr. Prout's valuable treatise, and as it is an extensive subject, our limits do not allow us to enter upon it. At page 329, paragraph 321, the following sentence occurs: "Nitric acid may be habitually discharged for years before that condition of kidney takes place which appears to dispose its concretion into a calculus." We presume that nitric acid is here a misprint for lithic acid.

Escharotics. In our opinion escharotics ought to have been a subdivision of epispastics; for the principles of action in rubefacients and blistering substances and escharotics, or those which are supposed to act chemically are nearly allied. This may be deduced from the fact, that the greater number of this order of medicines may be so regulated in strength as to produce all those different effects. A dilute solution of potass will cause inflammation; one more concentrated, an elevation of the cuticle by a serous fluid; but the solid caustic produces complete decomposition of this and other textures. The application of heat may also be so regulated as to produce nearly the same results. Dr. Paris states that during the employment of caustic potass as an escharotic, ammonia is generated by the combination of the hydrogen and nitrogen, and that it may be detected by inverting over the surface a small jar moistened with hydrochloric acid, when the fumes of hydrochlorate of ammonia become visible.

The observations on Mechanical Remedies and Anthelmintics present nothing novel.

Diluents. The author makes the following observations on the misapplication of this order of remedies:

"Dr. Davy found by experiment that when an animal is bled to death the last portions of blood are of a much lower specific gravity than those which flow at the commencement, in consequence of the former containing more water, which it is to be inferred was derived by the increased activity of the absorbents from the mucous and serous membranes. Since, then, venesection promotes and accelerates absorption (108), it is clear that in inflammatory diseases, where we have recourse to bloodletting in order to diminish the volume of the circulating fluids, we ought not to suffer the patient to indulge in an unrestrained use of liquids, which he eagerly demands to satisfy a thirst which is probably the natural consequence of an increased absorption. In such cases it is often better to administer liquids in small divided doses, which will have the effect of moderating the thirst without overloading the arterial system and bringing back that tension and plenitude which it has been our object to relieve. These views are equally important to the surgeon, for they will guide him in the treatment of patients who have undergone operations, for in some cases it will be desirable to restore as speedily as possible the blood that may have been lost, while in others it may be equally advisable to adopt an opposite practice." (pp. 359-60.)

This line of practice is problematical; for, on the one hand, the appeasing of thirst diminishes febrile excitement and promotes diuresis; while it is highly probable that the very dilution of the blood may tend to resolve inflammatory action, even although the blood-vessels should be redistended to their former caliber, which, in practice, however, we have seen no reason to believe. The following opinion is also questionable:

"It cannot be denied that exorbitant potation has a tendency to produce fat, which may depend upon the vascular distention, and consequently diminished absorption thus occasioned. It has been explained on the supposition that water yields hydrogen by its decomposition; but it has been already stated (26) that we have no reason to suppose that it is ever resolved into its elements in the living system." (p. 360.)

It is contrary to the modern theory of assimilation, and in our opinion contrary to experience, to believe that water containing no alimentary substance can tend to the production of fat except indirectly in the promotion of digestion. The same may be said of condiments.

Alteratives. These are restricted by the author to the preparations of mercury, which "increase the activity of the secreting organs and imperceptibly restore healthy action," &c.

Part III. *The Theory and Art of Prescribing.* The author commences this important part of his work by a comprehensive introduction, in which, after condemning the polypharmacy of the ancients, he animadverts upon the tendency to the opposite extreme, or an inactive simplicity. He alludes, with some point, to the combinations of nature, and the efficiency of some natural products when compared with the proximate principles produced from them. While discussing the objects to be attained by mixing and combining medicinal substances, the author gives the following elegant and powerful passage, to the discomfiture of empirical practitioners:

"Now, let me ask, what constitutes the essential difference between the true physician and his counterfeit, between the philosopher and the empiric? Simply this; that the latter exhibits the same medicine in every disease, however widely each may differ from the other in its symptoms and character, while the former examines, in a spirit of philosophic analysis, all the existing peculiarities of his patient and of his disorder, and being thus led by a sagacious induction to an estimate of his vital powers and to a knowledge of the nature and condition of the peccant organs, he graduates and adapts, with a sound discretion and with a correct judgment of his agents, such means as may be best calculated to control and correct their morbid condition." (p. 374.)

Five objects may be attained in the composition of formulæ; and these we shall notice in their order.

1. "*To promote the action of the basis or principal medicine.*" (p. 376.) The author considers it an important generalization that "a combination of similar remedies will produce a more certain, speedy,' and considerable effect than an equivalent dose of any single one." (p. 377.) That this law may hold in a number of cases we do not presume to question; but it has so many exceptions, in our opinion, that its claims to such a designation may with propriety be questioned. Exhilarants, excitants, or aromatic stimulants, are brought forward among others as examples:

"There are probably no remedies which receive greater mutual benefit by intermixture than the individuals which compose this class; for they not only thus acquire increased force and efficacy, but at the same time they lose much of their acrimony. If, for instance, any one spice, as the dried capsule of the capsicum, be taken into the stomach, it will excite a sense of heat and uneasiness; a similar effect will follow the ingestion of a quantity of black pepper; but if an equivalent quantity of these two stimulants be given in combination with each other, no such sense of pain is produced; but, on the contrary, a pleasant warmth is experienced and a general glow is felt over the whole body; and if a greater number of spices be joined together, the chance of pain and inflammation being produced will be still farther diminished." (p. 379.)

We question *in toto* the accuracy of the above statement; for we ourselves have often taken capsicum in conjunction with black pepper, along with alimentary matters, and never remarked that the hot qualities of either were in any degree abated. Indeed, the acridity of capsicum is with the greatest difficulty disguised by any other substance, whether stimulating or demulcent. Some of the illustrations alluded to are not fair examples of the combination of similar remedies, as in the confectio opii, confectio piperis nigri; for the additions to the primary ingredient are substances that possess different qualities. The composition of the different varieties of snuff is also brought to bear upon this question:

"The local action of stimulants would also appear to fall under the same law; and perhaps the origin of the popular custom, so long observed, of mixing together the varieties of snuff may thus receive a plausible explanation: certain it is that by such combination the harsh pungency of each will be diminished, and the odour rendered more mellow and agreeable." (p. 380.)

That the practice of mixing various kinds of snuff together is adopted and approved of by manufacturers of this article and connoisseurs in the nicotian art we doubt not; but that a mixture of good, bad, and indifferent kinds will form an agreeable variety is extremely doubtful. It may be generally predicated that two good varieties when mixed together will form an agreeable compound, the precise odour of which however can alone be determined by experiment; but if an inferior kind, or what amounts to the same thing, one not generally esteemed, be compounded with a favorite one, no man would be safe in prophesying a satisfactory result. A similar practice of mixing various qualities together is adopted by spirit-merchants and perfumers; but those which are approved of are the result of experiments nearly as precise as the formulæ of our pharmacopœias. In justice to the author, we must however admit that many of his examples furnish excellent proofs of the advantages resulting from the combinations of emetics, cathartics, diuretics, diaphoretics, &c. He mentions some exceptions, such as the combination of camphor and ammonia during fever, and the use of narcotics on particular occasions. He disapproves of the combination of squills, calomel, and digitalis, because the latter rather tends by its depressing powers upon the circulation to counteract the stimulating effects of the others. Farther and more accurate experiments are necessary to determine this knotty point; for many practitioners of experience are in the habit of exhibiting all the three medicines in combination with advantage.

The action of the basis is also promoted by combining it "with substances of a different nature and which do not exert any chemical influence upon it, but are found by experience to be capable of rendering the stomach or system or any particular organ more susceptible of its action." (p. 387.) In this division are noticed the superior diuretic powers of a combination of squills and calomel, the effects of antimony in quickening the operation of saline purgatives, of opium in increasing the sudorific powers of antimony, the operation of the bitartrate of potass in evacuating mucus, the increased power of the compound powder of jalap, the effects of colchicum in quickening the operation of aloes, &c. The beneficial effects of a combination of bitters with an alkali are also brought forward in illustration of the above law. May not this arise, to a great extent, from the neutralization of the acid which often exists in cases of dyspepsia?

"The relative sweetness of sugar when in different degrees of purity, depends upon the operation of the same law of combination; pure sugar, as Dr. M'Culloch has very truly observed, however paradoxical it may appear, is not so sweet as that which is impure. The sweetness of molasses compared with that of refined sugar is too well known to require more than to be mentioned; the vegetable matter, in this case, increases the effect of the saccharine principle with which it is combined." (pp. 391-2.)

We do not see the force of this reasoning. If molasses were the same kind of sugar as the refined, but disguised with vegetable impurities, the author's conclusion might be admitted; but as molasses is chiefly composed of uncrystallizable sugar its chemical qualities are different from those of the other, and why should its natural sweetness be not also different? According to Dr. Paris venesection increases the effects of cathartic medicines, calomel, opiates, &c. Purgatives awaken the susceptibility of the body to mercurial impressions, and to the effects of "steel medicines" and to diuretics: change of diet and habits are also *adjuvantia.*

2. "*To connect the operation of the basis by obviating any unpleasant effects it might be likely to occasion, and which would prevent its intended action, and defeat the objects of its exhibition.*" (p. 399.) This article is well worthy of careful perusal, and contains many useful observations; but we must refer the reader to the work itself.

3. "*To obtain the joint operation of two or more medicines.* A. By combining those substances which are calculated to produce the same ultimate effects, although by totally different modes of operation." (p. 409.) This law is illustrated by a combination of the infusion of senna with neutral salts, digitalis, potass as a diuretic, opium and ipecacuan as a diaphoretic, &c. B. "By combining medicines which have entirely different powers, and which are required to obviate different symptoms, or to answer different indications." This section includes a numerous group, from which we shall select a few instances of combination.

*Narcotics with mercurials.* Five grains of the extract of conium with one of calomel is mentioned as beneficial in chronic rheumatism. The combination of calomel with opium is not here mentioned, a preparation the value of which in resolving inflammations, particularly those of an acute character in serous tissues, is not equalled by any other. The opium serves at least two important purposes; first, by preventing the action of the mercury on the bowels, and, second, by hastening its operation on the system. In what manner the latter operation is produced has not been satisfactorily explained.

*Astringents with tonics.* "I will take this opportunity to remark," says the author, "that in superseding the preparations of the bark by the salts of quina, we deprive ourselves of any power that may be derived from the astringent principle (tannic acid), and there is reason to believe that its presence in the native combination heightens the tonic virtues of the alkaloid." (p. 414.) Berzelius's opinion is at the same time quoted in proof of the estimated value of the tannic acid as a constituent of bark. We are disposed to concur with this opinion, and feel satisfied that the salts of quina are too often substituted for the bark itself, or for what we consider an excellent preparation of it, a decoction made with diluted sulphuric acid.

*Astringents with narcotics.* In diarrhea, three valuable indications are obtained by the union of a narcotic, an astringent, and an antacid; the first diminishes the peristaltic motion of the intestines, the second restrains the serous evacuation, and the third neutralizes acidity. The mineral tonics, such as the sulphates of zinc and copper, may also be combined advantageously with opium.

*Purgatives with narcotics and antispasmodics.* In colica pictonum, opium is often highly beneficial, as it allays the spasm which is the chief obstacle to the action of the purgative. Aloes and assafetida or sagapenum, are useful in flatulent colic. The combination of some aromatic stimulant with drastic purgatives, is also of great service in preventing griping, particularly in that arising from aloes, colocynth, senna, gamboge, &c.

*Diuretics with excitants.* The author recommends a combination of squills, ammonia and ether, in cases where the system requires to be excited and upheld; and several formulæ are referred to. In such cases or in those where dropsical accumulations occur in weak or cachectic individuals, we have found a portion of gin or whisky a valuable adjuvant.

*Expectorants with excitants.* In peripneumonia notha, when the powers of life are ebbing and the lungs oppressed with viscid mucus, ammonia is recommended, to which small doses of opium may be added, if it does not check expectoration. In conclusion, the author justly remarks:

"In the construction of these composite formulæ, considerable experience and judgment are required. In the foregoing section I have endeavoured to point out, generally, the nature of the advantages to be obtained by such combinations, but they are not to be adopted without the nicest discriminations. No written instructions can ever embrace the whole extent of the subject; its leading points, however, may be seized by the student, and made the topics of his own reflections and examinations." (p. 420.)

4. "*To obtain a new and active remedy, not afforded by any single substance.*" (p. 422.) This is a dangerous field of practice and ought not to be adopted, in extemporaneous prescription, without careful examination and experiment. The decomposition of the sulphate of iron with carbonate of soda, resulting in the formation of the proto-carbonate of iron, an entirely new compound, is an exemplification of this law. Formula 90 (p. 604) is also adduced by the author, and it is the following; R. sodæ sesquicarb. ʒij, ferri sulph. gr. iij, magnesiæ carbonat. ʒj, aquæ Oss, acid. sulph. dilut. fʒx. In this case, sulphates of soda and magnesia are formed, holding in solution proto-carbonate of iron with excess of carbonic acid gas. In the development of active principles, the author alludes to a plaster, which is stated to be very efficacious in curing the swelling of the bursæ of the patella, a disease common to housemaids. It is composed of hydrochlorate of ammonia, soap and lead plaster; and the alkali of the soap slowly combines with the acid and liberates the ammonia. (Form. 17, p. 591.) The black and yellow washes are also mentioned as examples.

The increase or diminution of the solubility of substances also influences their operation. Dr. Paris endeavours to account for the varieties of action on the intestinal canal of gamboge, aloes, and colocynth, &c. upon their various degrees of solubility. Although there may be a portion of truth in his remarks, we are inclined to think that further and more accurate experiments are still required to determine these points. Mixtures of different saline purgatives are stated to be more active than an equivalent dose of any single one; as for example, the sulphate of magnesia and tartrate of potass and soda. The author attributes the griping effects of some purgatives to their comparative insolubility. This, a priori, would seem to be the case with resinous purgatives; but the same result is produced by an infusion of senna, buckthorn juice, &c. which proves that it also depends upon the irritating qualities of the agent.

The state of insolubility has a powerful effect on poisonous agents; thus many substances, such as the acetate of lead, are rendered inoperative by being thrown down in an insoluble form.

5. "*To afford an eligible form.*" (p. 441.) "It should ever be our object," says the author, "to accommodate, as far as we are able, the form and flavour of our medicines to the taste and caprice of the patient, whose prejudices should never fall coldly upon the ear of the physician; for such is their influence upon the body, that by a little address they may often be enlisted into our service, while by opposing them they are rendered formidable obstacles to our plan of treatment." (p. 442.) The disagreeable flavour of opium is disguised by storax and saffron. In solution, we have found a little acid and sweet spirits of nitre very serviceable for this purpose. The taste of alkaline solution is rendered less disagreeable by combination with beer or a bitter infusion. (Form. 169, p. 618.) Acids are sheathed by mucilage, and (it might have been added) sugar. Castor oil and balsam of copaiba are less nauseous, when formed into an emulsion by albumen ovi or mucilage. The addition of a little ammonia or aqua potassæ and syrup is very useful in preserving the emulsive form. Some medicines may be exhibited in the effervescent state with bicarbonate of soda and lemon-juice; but care must be taken that no decomposition ensue. Some substances require the addition of some agent to prevent the spontaneous changes to which they are liable. Thus sugar, which is found so useful in the preservation of animal and vegetable substances, has also been found essential in preventing the chemical changes which some mineral agents undergo. Iodide of iron and the proto-carbonate of iron are two remarkable examples in point. It ought to be observed, however, that dilute solutions of sugar are very liable to fermentation, and for this reason syrups should not be introduced into mixtures, which are not to be speedily used, particularly during warm weather. A small portion of an aromatic tincture, when added to mixtures, is very efficacious in retarding this process. Indeed the addition of a portion of alcohol to infusions and decoctions of vegetable substances or salts, containing a vegetable acid, is in many instances absolutely necessary to prevent decomposition.

Prescriptions. The subject of prescription is a very important one, and involves several grave considerations. Simplicity of construction, with a small or moderate amount of ingredients, should ever be regarded as the greatest desideratum; as there is more safety and efficiency in this practice than when there is a multitude of *bases*, *adjuvants*, and *corrigents*, which, like camp-followers, become a dead weight on the energies of the others. There is a tendency, however, among men of small comprehension, to place reliance upon little conceits of their own, which they generally dress up according to their own notions of taste and efficiency, and in this state are palmed upon their patients and sometimes on the profession as infallible prescriptions for the cure of certain forms of diseases. Such receipts ought to be narrowly scrutinised, and if made the subject of experiments, the trials should be made with caution and accuracy, so that their virtues may be corroborated, or their inefficiency exposed.

"'Superflua nusquam non nocent.' Let him cherish," says the learned author, "this maxim in his remembrance, and in forming compounds, always discard from them every element which has not its mode of action clearly defined, unless indeed, as we shall hereafter explain, a general and paramount experience shall have stamped upon it the authentic seal of experience . . . . . A medicinal formula has been divided into four constituent parts, a plan which will be found to admit of useful application to practice . . . . .

"1. The Basis, or principal ingredient.

"2. The Adjuvans; that which assists and promotes its operation.

"3. The Corrigens; that which corrects its operation.

"4. The Constituents; that which imparts an agreeable form.

"These several elements, however, are not all necessarily present in every formula, since many medicines do not require any addition to promote their operation; and the mild and tractable nature of others renders the introduction of any corrective unnecessary, while some again are in their nature so manageable as not to require the interposition of any vehicle or constituent." (pp. 449-50.)

DOSES OF MEDICINES. "It would appear," says Dr. Paris, "that powerful doses are disposed to produce local rather than general effects. Experience seems to prove that, in this respect, the effect of an internal remedy is analogous to that upon external impression; if violent, it affects more particularly the part to which it is directly applied, as pinching does that of the skin; whereas titillation, which may be said to differ from the former only in degree, acts upon the whole system, and if long continued would even occasion convulsions." (p. 453.)

We are glad to be supported by the authority of our author in the advocacy of moderate doses of medicines, as we are convinced that a prevailing evil in the practice of this country is *over-dosing.* In the case of chronic diseases this evil is carried to a truly frightful amount; and we do not hesitate to say that in a pretty large proportion of cases, the imaginary agency of the medicines in the hands of the homœopathists, leaving nature undisturbed, is less injurious and more successful than the energetic empiricism so often practised. The intercourse in recent times with the continental nations has tended to mitigate considerably this evil; but it yet rages, to a melancholy extent, among the disciples of the pure London school. We must not, however, be misunderstood, as proscribing large doses altogether. In their proper place they are absolutely necessary. The doses of medicines ought to be regulated by age, sex, temperament, habit, diet, profession, climate, and season, nature and duration of disease, time of the day, idiosyncrasy, &c.; but we refer to the work itself for full instructions on these matters.

The next article designated *chemical pharmaceutical errors,* exemplifies the various errors which are or may be committed by those who have an imperfect knowledge of chemistry, and is well worthy of perusal by those who wish to attain accuracy or elegance in writing prescriptions. The blunders committed in this department, even by otherwise highly respectable practitioners, are more frequent than they would have been if chemistry had held its due place in their education; for we hesitate not to say that this branch of medical education has been too lightly appreciated by the great body of practitioners. A most valuable table of *incompatible substances* is appended to this article. It is more copious than any hitherto published, occupying about forty pages of the work, includes many of the medicines found in our British pharmacopœias, and being accompanied with symbols is compressed into small bulk.

ON THE PARTICULAR FORMS OF REMEDIES. *Powders.* The following rule of the author applies generally to this form of medicine:

"I think it may be received as a general rule that extreme pulverization assists the operation of all substances whose active principles are not readily soluble; and that of compound powders whose ingredients require intimate intermixtures, whilst it certainly impairs the virtues of such as contain a volatile principle which is easily dissipated, or extractive matter which is readily oxidized." (p. 545.)

In the prescription of some saline bodies, it ought to be remembered that their mixture with other substances sometimes gives rise to a soft or liquid compound from the water of crystallization being differently arranged. Some are also apt to attract moisture from the atmosphere, and are thus apt to communicate the same tendency to those with which they are mixed. Pulverized squill root is peculiarly liable to this result, and we have seen specimens assume the consistency of diachylon plaster. All such powders ought to be kept in small bottles very tightly corked.

*Pills.* The preparation of pills is of far more importance than is generally imagined; for medicines are often prescribed in this form, as being more convenient and agreeable to the patient. They ought to possess, among other qualities, a proper consistency, and should not become mouldy or hard, when they require to be kept for some time. Conserve of roses is the excipient in many of the formulæ for pills in the pharmacopœias, and is generally well adapted for preserving them in a soft state, as it has an affinity for moisture. We have also found the addition of a little fixed oil very useful for this purpose, and when soap is not objectionable in a pill containing gum resins, it has a similar effect; as in the compound colocynth or gamboge pill. When pills become very hard, they ought not to be employed without being previously softened, as they are liable to pass through the bowels undissolved.* A convenient method of doing this is to moisten them with spirits or water the day previous to their use.

*Inhalations.* Remedies in this form are not in general much valued by the profession, and the tar-vapour of Sir A. Crichton as well as the chlorines and iodine of more recent date, are now rarely heard of. By these remarks we do not mean to depreciate the usefulness of these and other substances, in certain bronchial affections; but rather to show that the exaggerated expectations with which their introduction was accompanied, have not been realised. The temperature and hygrometric qualities of the atmosphere are certainly of great importance in many pectoral and other diseases; and were it possible to obtain the other essentials in promoting health as accompaniments of an artificial atmosphere, an important object would be thus obtained. This, we fear, can only be had, with all the requisite advantages, in a climate of nature's own formation. We can, however, by such means impregnate the system thoroughly with mer-

* We had once, now many years since, a ludicrous instance of this in our own practice, in consultation with an apothecary of the old school, whose pathology was also not very modern. Before seeing the patient we were told that it was a case of *gall-stones*, and on inquiry were further informed that though a *vast number* of these bodies had been passed, the patient had never suffered pain. This naturally surprised us, and our surprise was not lessened when the patient produced from the cupboard several pill-boxes, full of the said gall-stones! On even a slight inspection these were discovered to be pills, ("Boerhaave's red pill,") which the old gentleman, a toothless ecclesiastic, had been long in the habit of taking for a cutaneous affection!

cury and other agents, but the effects are often so uncertain as to render it by no means an eligible method.

*Blisters.* Dr. Paris does not notice the blistering *tissues* and *chartæ* of various chemists, which we think he might have done as an improvement of modern times, more particularly as it has been asserted that they produce no strangury. He is of opinion that blisters should not be kept applied, until vesication has occurred, as this effect generally follows the dressing. That this is a frequent result we do not question, but in the acute and dangerous internal diseases of adults, we are led by experience to believe that the blister is the most efficacious that is retained *in situ* until thorough vesication is produced. The opinions expressed in the following paragraph, are also very doubtful if not decidedly erroneous:

"The popular practice of copious potations to protect the kidneys from any anticipated irritation is wholly erroneous; and actually counteracts any benefit which might be derived from the action of a blister as an evacuant. (p. 357.) Erroneous views have also been entertained with regard to the effects of a blister bearing an exact proportion to its size. Large blisters, while they are far more efficient as evacuants, do not produce greater pain and constitutional irritation than those of smaller size." (p. 583.)

We are not in the habit of recommending copious potations, with the object of preventing strangury, for we are doubtful of their beneficial agency; but certainly we do not believe that this practice is productive of any very injurious effects, in inflammatory diseases, as we have endeavoured to show in a former part of this article. Although we approve of large blisters in certain affections, yet in our experience—and theory supports us—they always occasion more pain and irritation than small blisters; and we have often seen the system highly excited for a day or two by the former, whereas the latter more rarely produce much inconvenience.

The illustrative formulæ appended to the work are numerous and select, and being arranged under the various orders of medicines, with key-letters, &c. will be found of great use to the junior practitioner.

As we have now given a pretty copious analysis of Dr. Paris's work, we must here take leave of it and its learned and accomplished author. On several important subjects we have had occasion to differ in opinion from Dr. Paris, but from the prominent station of the writer, and the high character of the work, we considered it the more our duty to point out some of his more questionable dogmas and doctrines. The department of "Special Pharmacology" has been altogether omitted in the present edition; and there is sufficient internal evidence that the pharmacologia has been rewritten, and remodelled upon the theories and discoveries of modern times. Many of the chapters still contain a considerable amount of irrelevant, or at least very debateable matter. These portions of the treatise might, with great advantage to the reader, have been condensed, and the rules and principles confined to matters which were thoroughly established. The work, however, as a whole, is one of great erudition and great ability; it contains a large amount of information; it is elegantly and perspicuously written; and it will form an excellent guide to the practitioner, in compounding and prescribing the articles of the materia medica. We therefore recommend it in strong terms to the attention of all our readers.

